# Cecal Diverticulitis in a Geriatric Patient

**DOI:** 10.7759/cureus.28231

**Published:** 2022-08-21

**Authors:** Pooja Kanyadan, Carlos Lopez Ortiz, Rohan K Mangal, Thor S Stead, Latha Ganti

**Affiliations:** 1 Biology, Wheeler High School, Marietta, USA; 2 Emergency Medicine, HCA Florida Ocala Hospital, Ocala, USA; 3 Emergency Medicine, Envision Physician Services, Plantation, USA; 4 Emergency Medicine, University of Central Florida College of Medicine, Orlando, USA; 5 Medicine, University of Miami Miller School of Medicine, Miami, USA; 6 Medicine, Warren Alpert Medical School of Brown University, Providence, USA

**Keywords:** inflammation, small intestine, large intestine, cecal diverticulitis, emergency medicine

## Abstract

The authors present the case of a 70-year-old man with cecal (right-sided) diverticulitis. Cecal diverticulitis is frequently confused with appendicitis, which could potentially lead to unnecessary intervention. Cecal diverticulitis is usually seen in the fourth decade of life, whereas the patient who presented was significantly older. The risk factors, clinical presentation, imaging findings, and emergency department management are discussed.

## Introduction

Cecal diverticulitis occurs when a small pouch at the cecum, the junction between the small and large intestines, becomes inflamed and infected. The cecum sits on the lower right side of the abdomen, and the symptoms of cecal diverticulitis are often mistaken for appendicitis or irritable bowel syndrome. This is because diverticular disease typically occurs within the sigmoid colon, the portion of the large intestine closest to the rectum [1]. Diverticular disease of the sigmoid colon usually presents with pain in the left lower abdomen. Cecal diverticulitis is rare given the cecal diameter is large, whereas in the left descending colon and sigmoid, the caliber is smaller and thus less accommodating to pressure. It has been reported that cecal diverticula comprise 3.6% of all colonic diverticula [2]. Because cecal diverticulitis is so rare in the elderly patient, this pathology can often be mistaken for acute appendicitis, acute mesenteric ischemia, nephrolithiasis, or gall bladder disease. CT imaging of cecal diverticulitis reveals the presence of a thickened and/or inflamed cecal diverticulum [3]. In this case report, the authors describe the case of a patient who presented to the emergency department complaining of abdominal pain and constipation.

## Case presentation

A 70-year-old male with a past medical history of insulin-dependent type 2 diabetes, hypothyroidism, dyslipidemia, hypertension, coronary artery disease, and a prior surgical history of cholecystectomy presented to the emergency department (ED) complaining of abdominal pain and constipation. His abdominal pain began the previous evening, and it was associated with several days of constipation. His pain was not triggered by any specific activity and was progressively increasing in intensity. The patient stated that he had not had a bowel movement for six days, for which he took over-the-counter laxative pills without improvement of symptoms. He also complained of having difficulty urinating and chills but denied any fever, nausea, vomiting, or diarrhea. He stated that he had episodes of constipation in the past. The patient's family history was positive for diabetes and heart disease. The patient denied smoking and any recreational drug use. His vital signs included a temperature of 98.0°F, blood pressure of 147/66 mmHg, heart rate of 62 beats per minute, respiratory rate of 18 breaths per minute, and oxygen saturation of 100% on room air.

His physical examination showed moderate right lower quadrant and suprapubic tenderness to palpation without guarding or rebound. Laboratory analysis revealed elevated creatinine and glucose. Calcium, albumin, hemoglobin, and hematocrit were decreased (Table 1).

**Table 1:** Patient’s laboratory values

Name of Lab	Reference Range	Value
Sodium	135 - 145 mmol/L	140
Potassium	3.5 - 5.3 mmol/L	4.9
Chloride	99 - 111 mmol/L	109
Carbon dioxide	21 - 32 mmol/L	26
Blood urea nitrogen	7 - 22 mg/dL	38
Creatinine	0.6 - 1.3 mg/dL	2.1
Glomerular filtration rate	> 60	30
Glucose	74 - 106 mg/dL	194
Calcium	8.4 - 10.2 mg/dL	8.3
Total bilirubin	0.0 - 1.0 mg/dL	0.2
Aspartate aminotransferase	7 - 37 units/L	16
Alanine aminotransferase	12 - 78 units/L	23
Total alkaline phosphatase	50 - 136 units/L	85
Total protein	6.4 - 8.2 g/dL	6.8
Albumin	3.4 - 5.0 g/dL	3.0
Lipase	65 - 230 units/L	114
White blood cells	4.0 - 10.5 10^3/uL	7.0
Red blood cells	4.63 - 6.08 10^6/uL	3.95
Hemoglobin	13.7 - 17.5 g/dL	11.6
Hematocrit	40.1 - 51.0 %	37.9
Platelet count	150 - 400 10^3/uL	235

**Figure 1:**
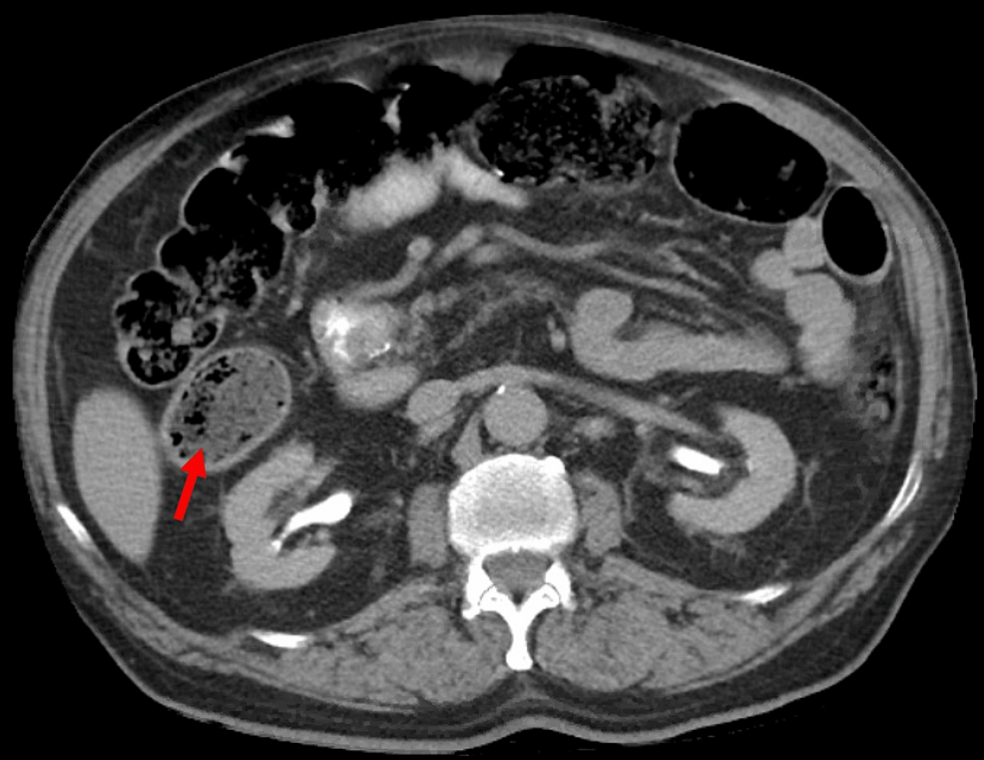
CT axial scan demonstrating cecal diverticulitis on the right (red arrow)

Figure 1 is an axial view CT scan of the abdomen and pelvis that revealed mild inflammatory changes involving the cecum, which suggested cecitis versus cecal diverticulitis, with no evidence of abscess (Figure 1).

**Figure 2:**
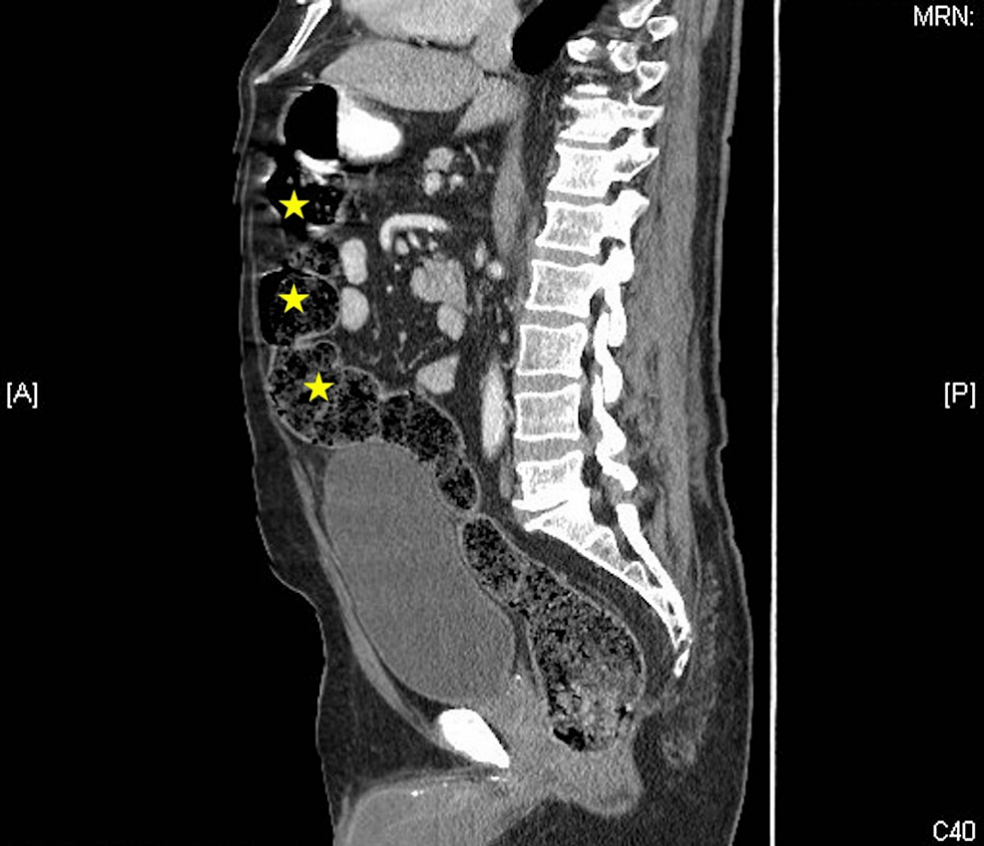
CT scan, sagittal view, demonstrating inflammatory changes surrounding cecal diverticula, consistent with diverticulitis (stars)

Figure 2 is the saggital view of the CT scan, again demonstrating cecal inflammation (Figure 2).

The patient was given intravenous ciprofloxacin and metronidazole and admitted to the hospital for further management. He was discharged on Day 3 with oral antibiotics.

## Discussion

**Figure 3:**
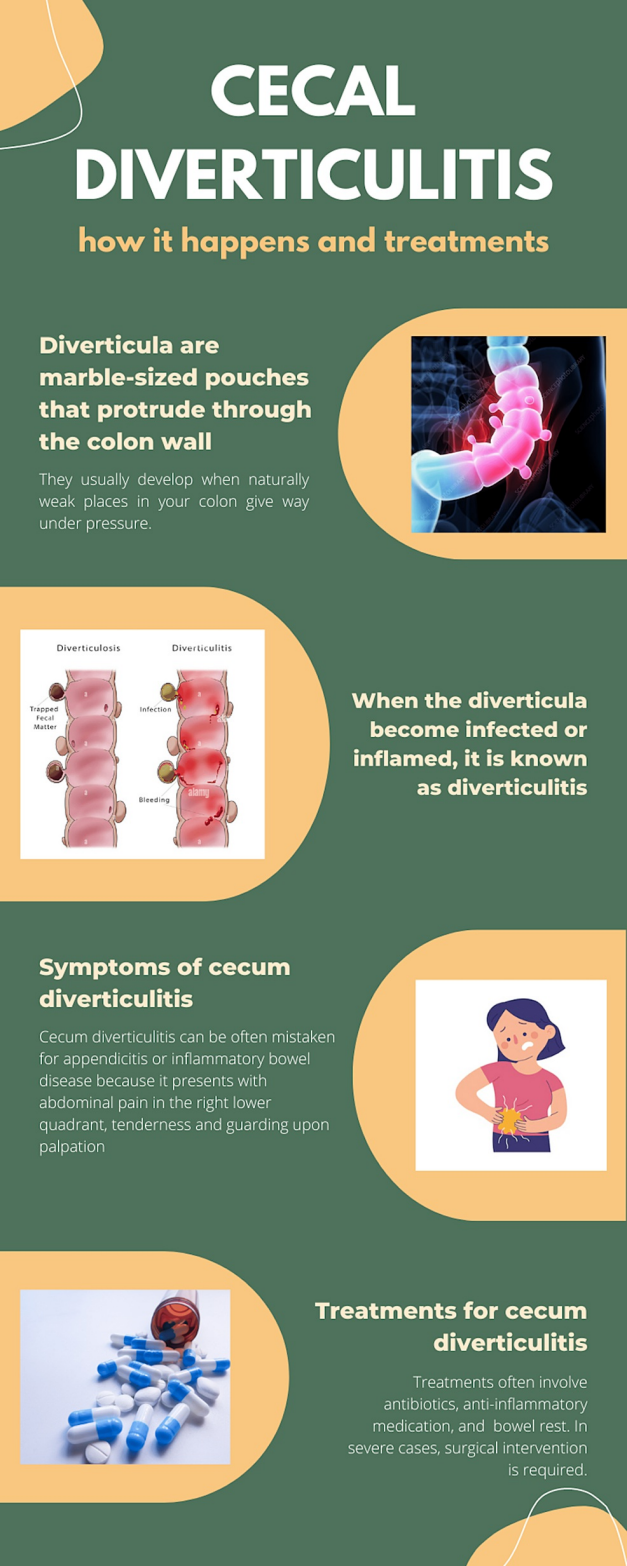
An illustration of cecal diverticulitis and its treatments Created by Pooja Kanyadan on Canva.com

The cecum is a pouch that sits in the lower right side of the abdomen and receives undigested food material from the small intestine [4]. It absorbs fluids and salts and mixes undigested food with mucus. Beneath the internal wall of the cecum is muscle tissue that aids in the digestion and movement of the undigested food material to the large intestine. Diverticula in the colon and/or the cecum most likely develop because of high pressure within these areas, and a diet low in fiber, aging, obesity, smoking, and lack of exercise can increase one's risk of developing diverticula [5]. These factors can weaken the walls of the colon and/or cause difficulty for undigested food material to move through the colon, which increases pressure within the walls of the colon and causes the diverticula to form. Over time, these bulges become inflamed, and sometimes, infected material gets stuck in them, causing diverticulitis. This case is unusual in that our patient was 70 years old. One would expect a sigmoid rather than cecal diverticulitis. Cecal diverticulitis presents mainly in younger patients with a median age of 44 years and predominantly occurs in males (Figure 3) [6].

Studies have shown that cecal diverticulitis affects more people of Asian descent compared to rates in Western countries. The development of cecal diverticula can lead to inflammation, infection, and rupture, so it is vital to diagnose and treat it immediately. Tests to diagnose this condition include blood tests and CT scans, but it is often discovered during laparoscopy when mistaken for acute appendicitis. Treatment of patients with cecal diverticulitis depends on whether the diverticulitis is complicated and on the co-morbidities of the patient. Hospitalized patients typically receive intravenous fluids, antibiotics, and anti-inflammatory medication. Oral food intake is restricted for an average of 24-48 hours. Although our patient had uncomplicated diverticulitis, he was hospitalized due to his co-morbidities. In acute cases where necrosis occurs on the diverticula, surgical intervention is required to remove dead tissue; however, this is uncommon. About 25% of people will develop complications relating to diverticulitis, including the formation of an abscess or blockage in the colon, an abnormal fistula developing between sections of the bowel, or peritonitis, which occurs when the diverticula rupture and spill intestinal contents into your abdominal cavity [7].

## Conclusions

This article discusses the evolution of uncomplicated cecal diverticulitis. The case is unusual in that cecal diverticulitis tends to occur in younger people, but the case presented occurred in a 70-year-old. Diverticulitis is a common condition that causes undue morbidity in patients. Understanding the lifestyle factors that can affect colon function is important for prevention. Excess pressure within the colon can lead to cecal diverticula and subsequent diverticulitis, as presented in this case report.
